# Embodied learning: introducing a taxonomy based on bodily engagement and task integration

**DOI:** 10.1186/s41235-018-0092-9

**Published:** 2018-03-07

**Authors:** Alexander Skulmowski, Günter Daniel Rey

**Affiliations:** Psychology of Learning with Digital Media, Chemnitz University of Technology, Straße der Nationen 12, 09111 Chemnitz, Germany

**Keywords:** Embodied cognition, Learning, Bodily activity, Task integration, Cognitive load

## Abstract

Research on learning and education is increasingly influenced by theories of embodied cognition. Several embodiment-based interventions have been empirically investigated, including gesturing, interactive digital media, and bodily activity in general. This review aims to present the most important theoretical foundations of embodied cognition and their application to educational research. Furthermore, we critically review recent research concerning the effectiveness of embodiment interventions and develop a taxonomy to more properly characterize research on embodied cognition. The main dimensions of this taxonomy are bodily engagement (i.e. how much bodily activity is involved) and task integration (i.e. whether bodily activities are related to a learning task in a meaningful way or not). By locating studies on the 2 × 2 grid resulting from this taxonomy and assessing the corresponding learning outcomes, we identify opportunities, problems, and challenges of research on embodied learning.

## Significance

The aim of this theoretical paper is to build a bridge between theoretical and applied advances in the field of embodied cognition (EC) research as it pertains to learning and education. To this end, we will present the major theoretical roots of current EC research, discuss whether embodiment approaches have been found to enhance learning processes in applied empirical studies, and offer an interpretation concerning the meaning of these findings for theoretical models. In addition, we aim to develop a taxonomy that can be used to classify the highly diverse implementations of EC in the field of learning and instruction.

## Introduction

Educational research incorporating findings from the research area of EC, often referred to as *embodied learning*, has established itself as an important field in the past few years (Lindgren & Johnson-Glenberg, 2013). EC is a research paradigm within the cognitive sciences describing how our body and our environment are related to cognitive processes (Barsalou, 1999; Beilock, 2015; Glenberg, 2010; Shapiro, 2010). Due to the growth of the field of embodied learning, a closer look at the different approaches to EC, their potential for educational settings, as well as current demonstrations of their effectivity is needed.

As we will present in the following, there is a wide variety of ways to transfer EC into learning to be found in the current literature (for a related discussion, see Skulmowski & Rey, 2017b). On the one hand, a large part of embodied learning research is concerned with instructional settings involving learners’ entire body (e.g. Johnson-Glenberg, Birchfield, Tolentino, & Koziupa, 2014; Lindgren, Tscholl, Wang, & Johnson, 2016). However, other researchers have focused on the potential uses of embodied phenomena besides full-body movement for educational contexts. These aspects include gesturing (for overviews, see Goldin-Meadow, 2011; Pouw, de Nooijer, van Gog, Zwaan, & Paas, 2014; Roth, 2001) or even minor implementations of EC such as assessing whether the display of human hands in animations can aid learning compared with disembodied arrow symbols (de Koning & Tabbers, 2013). Although some theoretical models emphasize the role of extensive forms of bodily movement (such as locomotion) in embodied learning research (e.g. Johnson-Glenberg et al., 2014), we propose a more general model based on the dimensions of bodily engagement and task integration. This taxonomy allows us to compare and discuss embodied learning studies ranging from only limited degrees of movement to full-body movement systematically and informatively (for similarly broad perspectives on educational EC research, see de Koning & Tabbers, 2011; van Gog, Paas, Marcus, Ayres, & Sweller, 2009). In this paper, we aim to develop such a taxonomy while reviewing recent literature on embodied learning. The following overview presents the most important theoretical origins of EC and emphasizes how many different types of research questions are included in the umbrella term of embodiment.

## Review

Several reviews and theory papers concerning the role of EC within the field of educational psychology introduced current findings from cognitive psychology and neuroscience to a wider audience within the field of educational and instructional psychology (e.g. de Koning & Tabbers, 2011; Paas & Sweller, 2012; van Gog et al., 2009). The following sections are aimed at illustrating the wide variety of approaches to EC that more applied fields focusing on learning and education currently utilize. A taxonomy of embodied learning will need to be able to categorize research drawing on all of these different aspects of EC.

### Embodiment and multisensory cognitive processing

One of the most influential theoretical approaches of EC has been Barsalou’s (1999) framework of *perceptual symbol systems*. This account suggests that humans use their sensory neural structures to create multisensory representations of their environment (for overviews on EC and language, see Pulvermüller, 2013; Zwaan, 2014). This thread of research revealed that humans reuse those brain structures that are active during perception when mentally imagining an object or action (Barsalou, 1999, 2003, 2008; for an overview on this aspect, see Anderson, 2010). Barsalou’s (1999) model explicitly breaks with the idea of Fodor’s (1975) abstracted forms of symbolic representations as a description of the human conceptual system (see Glenberg, Witt, & Metcalfe, 2013, for a contrast between abstract and embodied theories of cognition). Based on the embodied view of human cognition, educational researchers have started to develop interventions aimed at making learning contents easier to grasp by directly appealing to multisensory processing (for an overview, see de Koning & Tabbers, 2011). A variety of examples of EC-based interventions will be presented in the following sections.

### Gestures

Evidence for the importance of bodily action in the context of learning stems from gesture research (for reviews on gesture research, see Alibali, 2005; Pouw et al., 2014; for an overview of gesture research relevant to learning, see Goldin-Meadow, 2010). Experimental research conducted with young children is said to be a demonstration of the close relation between gesturing and language learning (Iverson & Goldin-Meadow, 2005). In the field of mathematics education, children were shown to benefit from observing teachers’ use of gestures as it increased the children’s inclination towards gesturing themselves (Cook & Goldin-Meadow, 2006). In that study, those children that performed gestures scored higher on a test (Cook & Goldin-Meadow, 2006). An explanation for the positive effects of gesturing may be an eased generation of knowledge structures in long-term memory compared to teaching methods relying solely on children’s verbalization (Cook, Mitchell, & Goldin-Meadow, 2008). Furthermore, a number of additional recent experiments have shown increases in learning outcomes when letting participants perform gestures (e.g. de Nooijer, van Gog, Paas, & Zwaan, 2013; Stieff, Lira, & Scopelitis, 2016; Toumpaniari, Loyens, Mavilidi, & Paas, 2015). Importantly, gesturing is not only relevant at a young age, but remains an important aspect of embodiment-based learning during later years (Kontra, Goldin-Meadow, & Beilock, 2012).

### Physical and virtual embodied learning

In addition to gestures, other forms of bodily activity have been investigated in the context of embodied learning. For instance, a significant theoretical component of EC theory is the notion of *enactment* (Gallagher & Lindgren, 2015). The bodily enactment of learning targets occurs when bodily movements are semantically related to these targets (see Hutto, Kirchhoff, & Abrahamson, 2015 and Gallagher & Lindgren, 2015, for overviews; see also de Koning & Tabbers, 2011). Educational researchers have begun to exploit learning strategies focusing on enactment in several ways (see Fiorella & Mayer, 2016, for an overview). One example discussed by Fiorella and Mayer (2016) are studies focusing on reading comprehension (such as Glenberg, Gutierrez, Levin, Japuntich, & Kaschak, 2004). As reading comprehension is considered to be related to EC (for an overview of this relation, see de Koning & van der Schoot, 2013), several studies demonstrated how reading comprehension can be enhanced when children physically perform a story they are reading (e.g., Glenberg, 2011; Glenberg et al., 2004).

A wide variety of research questions inspired by EC are investigated using digital learning media (for overviews, see de Koning & Tabbers, 2011; Lindgren & Johnson-Glenberg, 2013). For example, Pouw, van Gog, Zwaan, and Paas (2016) demonstrated that animations depicting learning content from the domain of physics can be enhanced by including a drawing of a human to help learners understand an otherwise abstract relation. Other examples include studies examining whether particular types of tablet computer interactions yield higher learning results (e.g. Agostinho et al., 2015; Dubé & McEwen, 2015) or whether interactive mixed reality settings involving bodily movement can offer advantages for learning (e.g. Johnson-Glenberg et al., 2014; Johnson-Glenberg, Megowan-Romanowicz, Birchfield, & Savio-Ramos, 2016; Lindgren et al., 2016).

### Taxonomies of embodiment in education

The reviewed literature suggests an enormous diversity of research questions and embodiment implementations when translated to learning and educational settings (see also Skulmowski & Rey, 2017b). Several taxonomies focusing on embodiment interventions in the context of education have been presented recently and will be described below.

Melcer and Isbister (2016) developed a framework encompassing several categories that enables comparisons between the design of embodied learning settings. Their categorization system is of a rather technical nature and was developed as a means to determine new combinations of embodied learning features for digital learning media (Melcer & Isbister, 2016). The seven main categories in the Melcer and Isbister (2016) system are: physicality; transforms; mapping; correspondence; mode of play; coordination; and environment. Each of these dimensions may be assigned different properties to categorize an embodied learning implementation (Melcer & Isbister, 2016). For instance, the dimension environment allows to categorize (components of) such an implementation as being developed as a virtual reality application, a mixed reality system, or as taking place in the non-virtual world (Melcer & Isbister, 2016).

Malinverni and Pares (2014) compiled a number of relevant categories to perform a systematic review of EC studies, including the theoretical context in which a study was performed, but also aspects of the user interface. Five main categories are listed by Malinverni and Pares (2014): theoretical framework; design strategy; educational context; interaction design; and evaluation. These categories contain additional subcategories (Malinverni & Pares, 2014).

Johnson-Glenberg et al. (2014) developed a taxonomy for educational EC research. Their taxonomy comprises three factors: motoric engagement; gestural congruency; and perceived immersion (Johnson-Glenberg et al., 2014). Johnson-Glenberg et al. (2014) emphasize the role of locomotion as a major contributor to motoric engagement. Furthermore, they define gestural congruency as the degree of how well a gesture matches a particular learning item (Johnson-Glenberg et al., 2014). Lastly, perceived immersion is understood as it pertains to virtual reality and related technologies (Johnson-Glenberg et al., 2014). Johnson-Glenberg et al. (2014) list specific combinations of these three factors that yield four distinct levels. They define the first level as non-interactive learning settings with materials being presented on a desktop computer or a tablet computer (Johnson-Glenberg et al., 2014). The next level introduces interactivity to the learning environment while the third level is reached when larger displays, full-body interactions (using motion-tracking devices), or both are integrated into an embodied learning setting (Johnson-Glenberg et al., 2014). The fourth and highest level of embodiment in learning settings requires learning environments to feature high degrees of bodily movement involving locomotion (Johnson-Glenberg et al., 2014).

However, Tran, Smith, and Buschkuehl (2017) have questioned the claim of a relation between these four embodiment levels defined by Johnson-Glenberg et al. (2014) and learning performance. We would like to expand this criticism of Tran et al. (2017) and identify additional weaknesses in the taxonomy proposed by Johnson-Glenberg et al. (2014): (1) considering the other reviewed taxonomies, we doubt that the combinations of the three factors motoric engagement, gestural congruency, and perceived immersion suggested by Johnson-Glenberg et al. (2014) are the optimal descriptive dimensions for educational embodiment; and (2) we consider the four embodiment degrees to be lacking in theoretical foundation.

Concerning the first issue, the three factors Johnson and colleagues (Johnson-Glenberg et al., 2014) propose have been the subject of several studies and have shown varying degrees of success in increasing learning performance (for a related criticism, see Tran et al., 2017). As we will present in more detail in the following sections, there have been studies in favor of bodily movement (e.g. Mavilidi, Okely, Chandler, Cliff, & Paas, 2015; Mavilidi, Okely, Chandler, & Paas, 2016; Ruiter, Loyens, & Paas, 2015) as well as studies arguing for more restrained instructional designs that offer only very basic interactions such as starting and pausing a simulation (e.g. Song et al., 2014). Concepts similar to the second factor, gestural congruency, have been presented as a contributor to the effectivity of embodied learning (Hald, de Nooijer, van Gog, & Bekkering, 2016; Hald, van den Hurk, & Bekkering, 2015). However, another study found no statistically significant differences regarding the accuracy in a transfer test between implementations that vary in regard to this factor (Pouw, Eielts, van Gog, Zwaan, & Paas, 2016). Therefore, one may argue that gestural congruency should be a factor to be considered in the design of embodiment interventions; yet, based on the reviewed results, we think that this aspect may not be informative enough to be used as a central classifier for EC research and should be revised. Lastly, while perceived immersion has been investigated in the context of embodied learning (e.g. Lindgren et al., 2016; see Dede, 2009, for an overview of immersion in the context of education), there are recent studies that did not lead to significantly higher learning scores when devices that offer higher degrees of immersion (as supposed by the authors of the studies) were used (e.g. Johnson-Glenberg et al., 2016; Johnson-Glenberg, Birchfield, Savvides, & Megowan-Romanowicz, 2011). However, it should be noted that these studies (Johnson-Glenberg et al., 2011, 2016) did not actually measure participants’ perceived immersion and are therefore based on the assumption that the different learning setups should, in theory, have led to different levels of immersion. Johnson-Glenberg et al. (2016) evaluated a mouse-controlled simulation, learning with a digital whiteboard, and a mixed reality learning setting. In addition, several of the embodied learning studies we reviewed earlier did not feature immersive digital environments but rather took place in ordinary instructional settings without the use of digital technology (e.g. Mavilidi et al., 2015; Ruiter et al., 2015). Hence, these scenarios cannot be distinguished informatively using the dimension of immersion proposed by Johnson-Glenberg et al. (2014). Thus, we conclude that immersion should not be regarded as one of the central factors for a taxonomy of embodiment research in the context of learning.

From our criticisms concerning the three dimensions of Johnson-Glenberg et al.’s (2014) taxonomy, we derive doubts concerning the logic behind the four levels of embodiment resulting from these three dimensions. As Tran et al. (2017) state, it is still to be determined whether a higher level of embodiment according to the four-level model presented by Johnson-Glenberg et al. (2014) necessarily entails a better learning performance. Furthermore, one may question why these four particular combinations should be the optimal way to categorize embodied learning research given the arguments we presented earlier.

With all the reviewed taxonomies, it is a matter of debate whether the particular dimensions of a taxonomy can be considered the most central and relevant properties of embodied learning settings. We consider taxonomies which include details concerning embodiment implementations (e.g. Malinverni & Pares, 2014; Melcer & Isbister, 2016) to be especially useful for educational technologists and related applied fields. The approach developed by Johnson-Glenberg et al. (2014) appears to be more appropriate for the analysis of educational embodiment research as it pertains to more basic building blocks of embodiment theory, such as the role of different types of movement (for an overview of the role of movement in EC, see Koziol, Budding, & Chidekel, 2012). Although the taxonomy developed by Johnson-Glenberg et al. (2014) enables us to assess whether bodily resources are involved in a learning setting, the semantic nature of actions, and whether the learning environment offers immersive qualities, we consider other factors to be at least as important for determining whether and how a learning task can be regarded as being influenced by EC.

### Integrated and incidental forms of embodiment

In order to describe an embodied learning setting in a meaningful way, we think that it is valuable to determine whether the intended form of embodiment is deeply *integrated* into the learning task or whether it is merely an *incidental* aspect. This approach follows the criticisms against a large part of EC research put forward by Wilson and Golonka (2013). Wilson and Golonka (2013) distinguish between two broad types of EC research. On the one side, Wilson and Golonka (2013) emphasize that EC research should consist of studies examining interactions between mental processes and their physical surroundings (including bodily capacities) as they pertain to the completion of tasks. On the other side, they present examples of experiments aimed at investigating how bodily influences can prime cognitive processes. Consequently, Wilson and Golonka (2013) consider the latter type of experiments to be of lower value for embodiment research than task-oriented studies testing hypotheses concerning the use of cognitive and physical resources. We define *integrated forms of embodied learning* to be aligned with Wilson and Golonka’s (2013) notion of task-related embodiment manipulations and *incidental forms of embodied learning* as examples of those studies that Wilson and Golonka (2013) describe as dealing with bodily priming effects. We will discuss examples of these types of studies below.

Research on embodiment often involves the manipulation of cognitive processes using incidental cues (e.g. Ackerman, Nocera, & Bargh, 2010), such as making information appear more important by presenting it on a heavy instead of a light object (e.g. Jostmann, Lakens, & Schubert, 2009). Such types of embodiment experiments have often been carried out in the context of judgment and decision-making (e.g. Ackerman et al., 2010), but more recently in the field of learning and metacognition (e.g. Alban & Kelley, 2013; Skulmowski & Rey, 2017a). For instance, Alban and Kelley (2013) were able to influence ratings concerning the ability to remember words by increasing the weight of the boxes that these words were attached to. Heavier boxes induced higher subjective ratings concerning one’s own ability to recall these words in later tests (Alban & Kelley, 2013). Skulmowski and Rey (2017a) extended this finding with studies indicating that wearing a heavy backpack during learning increased recall judgments and also heightened recall performance (at least when the learning contents were easy). Transferred to the field of education, we call such manipulations incidental forms of embodied learning.

On the other end of the spectrum, we consider studies in which embodiment aspects are connected inseparably with a learning task based on Wilson and Golonka’s (2013) task-oriented view of embodiment. For instance, this can entail comparisons between learning settings built around bodily activities compared with those enabling learning without requiring motor activity (e.g. Johnson-Glenberg et al., 2014; Song et al., 2014) or presenting information for multiple sensory modalities compared with only providing information for one modality (e.g. Skulmowski, Pradel, Kühnert, Brunnett, & Rey, 2016). We call such manipulations integrated forms of embodied learning (see Mavilidi et al., 2015, for a recent study and literature review on integration in embodied learning). Mavilidi et al. (2015) operationalized, among other conditions, the factor of integration by comparing a language learning intervention that lets children bodily enact foreign language words with merely exerting physical activity with a comparable degree of effort. Crucially, Mavilidi et al. (2015) demonstrated that an integrated physical learning task leads to higher learning performance than letting learners perform bodily exercises without a relation to the learning contents. A similar result was obtained in a study by Brooks and Goldin-Meadow (2016) that found an advantage for content-related movements over unrelated movements when teaching children mathematics (for an overview of the aspect of meaning-congruency in the context of embodied cognition, see Hald et al., 2016). These results clearly demonstrate that task integration is an important factor for embodied learning.

The factor of task integration bears some resemblance to the factor gestural congruency as defined by Johnson-Glenberg et al. (2014). A highly integrated form of embodiment and an implementation featuring a high gestural congruency would both exhibit a semantic relationship between a bodily activity and learning targets. However, the concept of task integration with a spectrum ranging from integrated to incidental is more general and thus can be applied to more types of embodiment research than gestural congruency. For instance, the embodiment manipulations found in some of the reviewed studies operating on incidental bodily cues (Alban & Kelley, 2013; Skulmowski & Rey, 2017a) can be categorized in a more informative way as examples of incidental embodied learning variants instead of merely referring to them as having a low gestural congruency (or none at all) in Johnson-Glenberg et al.’s (2014) model. Furthermore, categorizing studies using the dimension of task integration pertains to the more global aspect of how a study is designed rather than how a learning setting is used to implement embodiment. Thus, we suggest using Johnson-Glenberg et al.’s (2014) dimension of gestural congruency when discussing the concrete implementation(s) of embodiment aspects related to semantic relationships within study designs but argue for a distinction between integrated and incidental forms of embodiment when comparing study designs at a more abstract level.

In contrast to incidental forms of embodiment, integrated forms of embodiment can pose additional challenges when designing embodiment experiments (for an overview, see Wilson and Golonka, 2013). A crucial factor in ensuring a high internal validity of such experiments is the appropriate choice of control groups (see Castro-Alonso, Ayres, & Paas, 2016, for a related criticism of research in the field of multimedia learning). Educational embodiment researchers tend to perform research using widely available devices, such as video game consoles (e.g. Pouw, van Gog, et al., 2016) and tablets (e.g. Agostinho et al., 2015), which potentially puts limits on the amount of control experimenters have over the learning task. In some instances, studies rely on several different devices in order construct experimental groups aimed to assess the effects of different levels of embodiment on learners (e.g. Johnson-Glenberg et al., 2016). Under such circumstances, care must be taken when extrapolating conclusions due to confounding factors (Castro-Alonso et al., 2016; Rey, 2010).

### Bodily engagement

As the second dimension of our taxonomy, we propose to include *bodily engagement*. Bodily engagement and related notions of motor activation have been proposed as major characteristics of (educational) embodiment research in existing classification systems (e.g. Clifton et al., 2016; Johnson-Glenberg et al., 2014, 2016). We prefer the more general term bodily engagement instead of the term *motor engagement* introduced by Johnson-Glenberg et al. (2014) since it includes aspects of embodiment that lie beyond the nervous system (for overviews on environmental aspects of cognition, see Clark, 2008 and Wilson, 2002).

As Johnson-Glenberg et al. (2014) divide their scale of embodiment into four levels, they assign each of these four levels specific ranges of motor engagement. The first two levels of their embodiment model only feature restricted levels of motor engagement allowing learners to watch animations (first level) or to interact with simulations (second level) using desktop computers or tablets while remaining seated (Johnson-Glenberg et al., 2014). For the factor bodily engagement in our taxonomy, we would consider such forms of embodiment as *lower levels of bodily engagement*. Research on observing gestures and other human movements has been linked to embodiment research in the context of mirror neuron activity (de Koning & Tabbers, 2011; see also van Gog et al., 2009) and may be regarded to exhibit a similar level of bodily engagement that Johnson-Glenberg et al. (2014) define as their first level of embodiment. Many recent examples of gesture research (e.g. de Koning & Tabbers, 2013; Post, van Gog, Paas, & Zwaan, 2013) would fall into the first or second level of embodiment of Johnson-Glenberg et al.’s (2014) taxonomy. The third and fourth levels of embodiment in the model of Johnson-Glenberg et al. (2014) encompass letting learners perform bodily movements and locomotion, respectively. We define forms of embodiment qualifying for these two levels to be *higher levels of bodily engagement* in our taxonomy.

Several recent studies paint a positive picture concerning the inclusion of high levels of bodily engagement into learning tasks (e.g. Johnson-Glenberg et al., 2014; Lindgren et al., 2016; Mavilidi et al., 2015, 2016; Ruiter et al., 2015). Some of these studies focus on the effects of instructional activities incorporating walking and report positive results of movement-based interventions compared with teaching methods lacking bodily involvement (e.g. Johnson-Glenberg et al., 2014; Ruiter et al., 2015). However, there are studies demonstrating only small benefits of EC-based instruction featuring high bodily engagement. For instance, Johnson-Glenberg et al. (2016) could not find a significant overall learning advantage for higher embodiment levels that included a higher gestural congruency and in some instances a higher degree of sensorimotor engagement according to a taxonomy based on Johnson-Glenberg et al. (2014). Yet, Johnson-Glenberg et al. (2016) were able to detect higher knowledge retention with a delayed test for those participants who were assigned to an implementation involving a higher level of embodiment.

Various recent studies featuring minimal forms of bodily engagement have focused on the effects of basic interaction patterns involving hand movements and finger tracing on learning. Several studies support the idea that performing tracing activities with fingers and other simple hand movements can aid learners (e.g. Agostinho et al., 2015; Brooks & Goldin-Meadow, 2016; Dubé & McEwen, 2015; Ginns, Hu, Byrne, & Bobis, 2016; Hu, Ginns, & Bobis, 2015; Ouwehand, van Gog, & Paas, 2016). Results such as these can be taken as evidence for the claim that even very minor changes in interaction design towards bodily engagement can affect learning performance (see Schwartz & Plass, 2014, for a similar conclusion). Other research has revealed that interactivity can be more effective than merely letting learners observe an interaction (Jang, Vitale, Jyung, & Black, 2017) as well as having positive effects on working memory and affective variables (Vallée-Tourangeau, Sirota, & Vallée-Tourangeau, 2016; see also Domagk, Schwartz, & Plass, 2010, for a review on interactivity). Contrary to these results, some researchers highlight the pitfalls of interactivity (e.g., Song et al., 2014) and others assume that there may be ideal levels of interactivity (Kalet et al., 2012). Kalet et al. (2012) demonstrated that a medical simulation involving a restrained extent of interactivity in which mouse clicking on instructionally relevant items would start instructional animations leads to better learning outcomes than versions with less interactivity (i.e. only being able to start and stop animations) or more interactivity (i.e. being able to virtually enact medical procedures by moving the mouse). Song et al. (2014) presented the results of a study that compared four versions of a medical simulation illustrating how a stroke is generated in the brain. Song et al. (2014) specifically refer to embodiment literature such as Barsalou (2008) when discussing why stronger forms of activity might increase learning performance. The results of their study indicated that versions featuring only minor forms of interactivity (such as merely watching a simulation) actually lead to a better learning performance than versions demanding participants to control individual elements of the simulation using the mouse (Song et al., 2014).

Another form of rather low bodily engagement occurs if an embodied learning setting offers learners an opportunity to observe movements instead of performing movements (e.g. Brucker, Ehlis, Häußinger, Fallgatter, & Gerjets, 2015). Such studies are often conducted within a theoretical framing based on EC research in the case that learners’ attention is focused on a human model (for theoretical overviews, see de Koning & Tabbers, 2011 and van Gog et al., 2009). Thus, despite their low bodily engagement, we consider such studies as examples of embodiment research as they illuminate our understanding of the role of human movement in learning (see de Koning & Tabbers, 2011, for a similar perspective that includes minimal bodily movement). Letting learners observe human movements has generally shown positive effects on learning compared with more static formats of instruction (e.g. Brucker et al., 2015; Castro-Alonso, Ayres, & Paas, 2015; Fiorella & Mayer, 2016; Rueckert, Church, Avila, & Trejo, 2017) or with non-human movements (Pouw, van Gog, et al., 2016) in recent studies. Some studies have revealed moderating factors or boundary conditions of presenting movements, such as the congruency between the learners’ perspective and the perspective depicted in the learning materials (Fiorella, van Gog, Hoogerheide, & Mayer, 2016), learners’ gender (Wong, Castro-Alonso, Ayres, & Paas, 2015), and the depicted movement type (van Wermeskerken, Fijan, Eielts, & Pouw, 2016). A small number of recently published studies brought about results indicating no effects for observed movements (e.g. Ouwehand, van Gog, & Paas, 2015). For instance, attempts to enhance geometry learning by presenting participants with recorded eye movements in an effort to guide their attention did not lead to significant learning advantages (van Marlen, van Wermeskerken, Jarodzka, & van Gog, 2016).

The studies reviewed in this section underline that the concept of embodiment is used very broadly within the field of educational research (see Skulmowski & Rey, 2017b, for a similar conclusion). In many cases, educational researchers set out to test specific embodiment hypotheses by varying details in the presentation of learning contents or in interaction designs. Although many studies are not concerned with elaborate patterns of full-body motion, they still should be considered as equally important demonstrations of embodiment effects. Therefore, we see no reason why such studies should be considered to deal with a “less embodied” research question than experiments involving full-body activity (cf. Johnson-Glenberg et al., 2014). Rather, a taxonomy of embodied learning should also encompass research testing embodiment hypotheses that do not primarily focus on extensive bodily movement during learning, but embrace bodily engagement in more restrained forms. Intriguing examples for such interventions deal with cognitive offloading (i.e. using the environment to reduce cognitive demands; Kirsh & Maglio, 1994; Wilson, 2002; see Risko & Gilbert, 2016, for an overview). Interactions between learners and their environment have recently gained attention within the field of educational psychology (Choi, van Merriënboer, & Paas, 2014). It is important to note that cognitive offloading does not require elaborate interactions or a high degree of bodily activity, as some of these studies investigate rather basic questions such as under which circumstances humans prefer methods of non-mental information storage using pen and paper (Risko & Dunn, 2015). Certainly, this broad definition of bodily engagement should not be misunderstood in a manner suggesting that virtually all forms of cognitive learning research could somehow be construed to qualify as embodied learning research.

More generally, our review does not support the idea that higher levels of bodily engagement will in all cases lead to better learning outcomes than instructional designs featuring lower bodily involvement (see Tran et al., 2017, for a similar conclusion). In fact, a number of studies (Post et al., 2013; Ruiter et al., 2015; Skulmowski et al., 2016) warn of heightened cognitive demands stemming from linking too many EC-based interventions at once and identify unnecessary cognitive load (which these studies refer to as extraneous load following the model of cognitive load theory, Sweller, 1988; Sweller, van Merrienboer, & Paas, 1998) as a major risk for embodied learning. A study investigating movement patterns in the context of dance training showed advantages for rehearsing with simplified dance moves of a to-be-performed dance (Warburton, Wilson, Lynch, & Cuykendall, 2013). Similarly, other studies argue for less (complex) activity during learning in order to save cognitive capacities (e.g. Kalet et al., 2012; Song et al., 2014).

However, embodiment manipulations using very subtle bodily cues run the risk of not finding substantial effects on performance measures such as recall tests. For instance, Alban and Kelley (2013) conducted a series of studies aimed at biasing metacognitive judgments and recall results using weight cues that were relatively light. The studies featured a word-learning task in which words were attached to small boxes that differed regarding their weight (Experiments 2 to 4 in Alban & Kelley, 2013) or in which clipboards used to write down metacognitive judgments differed in their weight (Experiment 1 in Alban & Kelley, 2013). While they found significant differences regarding metacognitive judgments concerning recall performance in favor of words written on heavier items, no significant recall differences could be observed (Alban & Kelley, 2013). Alban and Kelley (2013) discuss that stronger differences in weight may be required in order to find differences in memory performance. In a similar study design involving more pronounced weight differences induced by having the experimental group wear a backpack, significant differences regarding metacognitive judgments as well as memory performance could be found (Skulmowski & Rey, 2017a). In line with the explanations given by Alban and Kelley (2013) and Skulmowski and Rey (2017a), the difference in the result pattern between the minor weight manipulation (Alban & Kelley, 2013) and the greater weight difference (Skulmowski & Rey, 2017a) could be attributed to the difference in the degree of bodily engagement (rephrased in the terminology of our taxonomy). Thus, arranging studies along the dimension of bodily engagement may help to compare the magnitude of different embodiment effects. Moreover, thinking of embodiment manipulations in this manner may improve the estimation of effect sizes and calculation of sample sizes (see Rabelo, Keller, Pilati, & Wicherts, 2015, for an overview of EC research and sample sizes). Judging from the differences between the two discussed examples of low task integration (Alban & Kelley, 2013; Skulmowski & Rey, 2017a), studies featuring lower bodily engagement will potentially have a lower statistical power than studies with higher degrees of bodily engagement (at least for particular types of measures).

Moreover, Skulmowski and Rey (2017b) compiled several measures of cognitive load that can be used in embodied learning studies, such as behavioral, physiological, and metacognitive measures. Johnson-Glenberg and Megowan-Romanowicz (2017) emphasize that the nature of a learning test can greatly affect how well advantages of an embodied learning implementation can be detected. In their study, the benefits of a more embodied learning mode only became noticeable using an embodied learning test involving gestures (Johnson-Glenberg & Megowan-Romanowicz, 2017). Furthermore, Skulmowski and Rey (2017b) recommend the use of repeated-measures designs when measuring cognitive load in educational studies involving embodiment.

### Summary

**Fig. 1:**
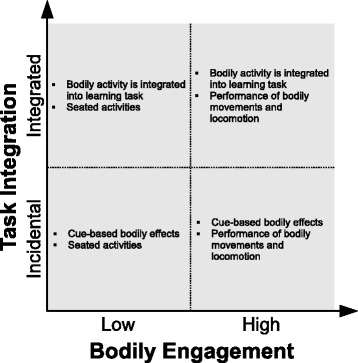
The 2 × 2 grid resulting from the proposed taxonomy presented in the section “Taxonomies of embodiment in education.” The four quadrants correspond to combinations of the two dimensions bodily engagement (low vs high) and task integration (incidental vs integrated). Low bodily engagement in our taxonomy is comparable to the lower two levels of embodiment defined by Johnson-Glenberg et al. (2014), i.e. watching animations or other seated interactions. Correspondingly, high bodily engagement in our taxonomy is comparable to the higher two levels of embodiment defined by Johnson-Glenberg et al. (2014), i.e. the performance of bodily movements and locomotion. Incidental embodiment manipulations aim to influence cognitive processes using cues (for an example, see Jostmann et al., 2009), while we define integrated forms of embodied learning to feature bodily activity integrated into a learning task (based on the task-oriented view of embodied cognition presented by Wilson and Golonka, 2013)

The reviewed literature and our classification of these studies along the dimensions of task integration and bodily engagement allow us to formulate recommendations concerning the design of embodied learning settings. The taxonomy results in a 2 × 2 grid with the dimensions task integration (incidental vs integrated) and bodily engagement (low vs high) and is presented together with criteria for the inclusion into one of the four quadrants of the grid in Fig. 1. Although the diagram in Fig. 1 appears to sharply divide the four combinations of factors, the dimensions should be regarded as continuous and the case may be made for regarding the boundaries as fuzzy; leading to intermediate forms of task integration and bodily engagement.

Research meeting the criteria for any of the four quadrants is faced with possibilities and challenges unique to each quadrant. Studies relying on low bodily engagement and incorporating only incidental embodiment manipulations may result in weak effects on some performance measures (e.g. Alban & Kelley, 2013). However, increasing the degree of bodily engagement can in some cases remedy this problem (e.g. Skulmowski & Rey, 2017a). A low degree of integration in itself may lead to worse learning results than an integrated intervention (Mavilidi et al., 2015). Turning to the dimension of bodily engagement, a large number of studies describing interventions with a lower level of bodily engagement, such as observing movements (e.g. Brucker et al., 2015) or performing gestures (e.g. de Nooijer et al., 2013), report successful outcomes. On the other hand, high bodily engagement has both been linked to learning gains (e.g. Johnson-Glenberg et al., 2016; Lindgren et al., 2016) as well as to the risk of cognitive overload (e.g. Ruiter et al., 2015; Skulmowski et al., 2016; Song et al., 2014). Some researchers have defined a medium degree of interactivity to be best suited for increasing learning performance (Kalet et al., 2012).

To conclude, the reviewed literature and taxonomy underline that neither should the degree of bodily involvement be used as an indicator of how “embodied” a form of instruction is (cf. Johnson-Glenberg et al., 2014) nor can it be expected that increases in bodily engagement automatically entail increases in learning performance (see Tran et al., 2017, for a related discussion).

## Conclusion and outlook

The proposed taxonomy of educational embodiment research highlights the possibilities and challenges involved in translating basic embodiment research into application. The two dimensions for EC research, task integration and bodily engagement, can be used to distinguish embodiment interventions on a theoretical level while also providing guidance for instructional designers aiming to apply EC findings. As we have presented in this review, bodily engagement should not be regarded as the primary dimension of embodied learning research, but also how strongly various degrees of bodily engagement are integrated into a task (based on Wilson and Golonka, 2013). Furthermore, the two dimensions we propose can be used in conjunction with one or more additional factors to describe subfields of educational research. More generally, the taxonomy and review presented here offer researchers from more basic fields insights into the findings and challenges within more applied fields of embodiment research.

## Abbreviations

EC: Embodied cognition
